# The Multivariate Largest Lyapunov Exponent as an Age-Related Metric of Quiet Standing Balance

**DOI:** 10.1155/2015/309756

**Published:** 2015-05-12

**Authors:** Kun Liu, Hongrui Wang, Jinzhuang Xiao

**Affiliations:** ^1^School of Electrical Engineering, Yanshan University, Qinhuangdao 066004, China; ^2^College of Electronic and Information Engineering, Hebei University, Baoding 071000, China

## Abstract

The largest Lyapunov exponent has been researched as a metric of the balance ability during human quiet standing. However, the sensitivity and accuracy of this measurement method are not good enough for clinical use. The present research proposes a metric of the human body's standing balance ability based on the multivariate largest Lyapunov exponent which can quantify the human standing balance. The dynamic multivariate time series of ankle, knee, and hip were measured by multiple electrical goniometers. Thirty-six normal people of different ages participated in the test. With acquired data, the multivariate largest Lyapunov exponent was calculated. Finally, the results of the proposed approach were analysed and compared with the traditional method, for which the largest Lyapunov exponent and power spectral density from the centre of pressure were also calculated. The following conclusions can be obtained. The multivariate largest Lyapunov exponent has a higher degree of differentiation in differentiating balance in eyes-closed conditions. The MLLE value reflects the overall coordination between multisegment movements. Individuals of different ages can be distinguished by their MLLE values. The standing stability of human is reduced with the increment of age.

## 1. Introduction

An age-related trend of a decline in human standing balance ability is obvious [1]. The method of effectively measuring the body's balance is significant for rehabilitation and diagnosis of the diseases of standing balance disorder [2]. In order to solve the corresponding actual application problems, some researchers have studied the relevant variables using mathematical models to simulate the human biomechanical or control system. Although some researchers have obtained valuable findings, it is impossible to precisely represent the human body, which is a complex biological system, by establishing a mathematical model [2].

Based on chaos theory, the largest Lyapunov exponent (LLE) can be used to understand the hidden properties of standing balance. LLE is a typically nonlinear parameter used to quantify the sensitivity to the initial conditions. It indicates the average rate of divergence of two neighbouring attractor trajectories. The existence of a positive exponent for almost all initial conditions in a bounded dynamic system is a widely used definition of deterministic chaos. A negative exponent implies that the orbits approach a common fixed point. Some researchers reconstructed the phase space of the standing centre of pressure (COP) data and then calculated the LLE. As all the LLE values were positive and close to 0, they suggested that the body's standing balance system is weakly chaotic [3, 4].

In previous studies, standing balance is evaluated by the COP trajectory from a force platform. However, the COP trajectory has its limitations because the human body is multisegmental and does not always act as a single inverted pendulum. Those approaches are not sensitive enough to investigate the dynamics of the system [5, 6]. By using multisensor systems, we can acquire information on human motion types, such as the flexion angle and body shaking acceleration time series.

In reality, because the time series obtained are of finite length and have noise mixed in, the phase space reconstruction of single-dimensional time series cannot always accurately describe the trajectory of the original dynamical system [7]. There has been a lot of research on multidimensional time series phase space reconstruction techniques [8]. Reference [9] proposed the multivariate largest Lyapunov exponent (MLLE) calculation method.

In this study, a metric of the human standing balance ability based on the MLLE is proposed. The dynamic multivariate data of ankle, knee, and hip can be measured by three joint angle sensors. Thirty-six normal people of different ages took part in the test, and the multivariate largest Lyapunov exponent was calculated from their time series. In order to statistically analyse and compare the proposed approach and the traditional method, the single variety LLE from the COP was also calculated. The results show that the MLLE is advantaged in differentiating balance under eyes-closed conditions; sway data are analysed especially under the eyes-closed condition, and this approach based on multidimensional time series confers significant advantages.

## 2. Methodology

### 2.1. Subjects

Thirty-six normal subjects of different ages and having no muscle or neurological movement disorders participated in the experiment. The subjects were divided into an elderly group and a young group according to age. The old group included 16 healthy old residents (8 females and 8 males; age range: 60–78 years; mean age: 65.7 ± 6.1 years; mean height: 161.6 ± 8.3 cm; average weight: 53.6 ± 9.3 kg); the young group included 16 healthy students (8 females and 8 males; age range: 22–33 years; mean age: 25.7 ± 3.1 years; mean height: 165.6 ± 6.8 cm; average weight: 60.6 ± 8.7 kg).

### 2.2. Sensors System

#### 2.2.1. Electrical Goniometer (Noraxon DDTS)

**Figure 1:**
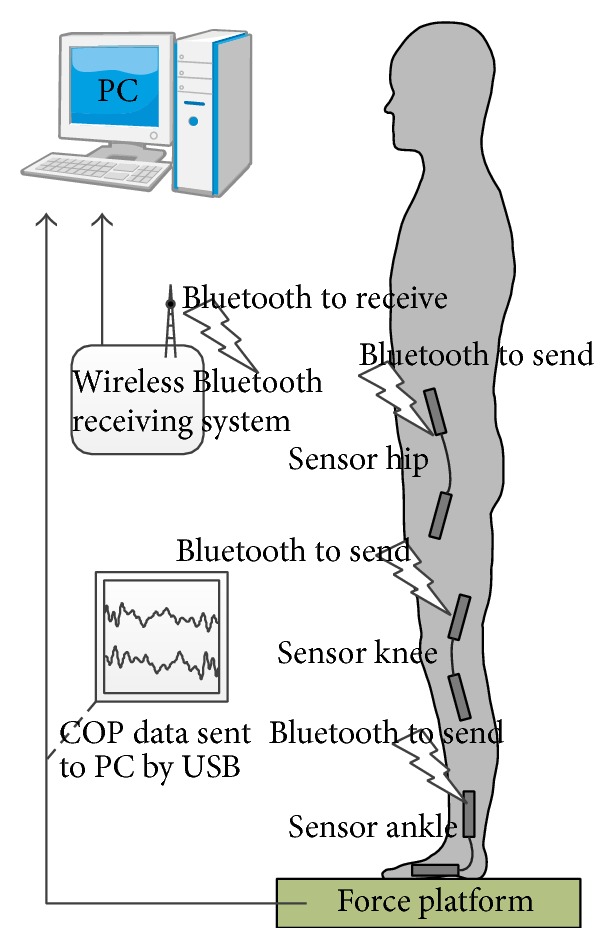
Posture of the subject and testing system. The angle sensor was positioned at the ankle, knee, and hip joints. Subjects stood barefoot on a force platform, with hands naturally at their sides. Their two feet were apart at their shoulder-width. Subjects were asked to stand upright with eyes-closed and eyes-open, respectively, for 100 s.

Three goniometers, which are attached on the hip, knee, and ankle joints (Figure 1), are used to collect the sagittal kinematics of the joints. The collected signal is sent to personal computer by Bluetooth.

#### 2.2.2. Force Plate

An OPT400600 (AMTI) is used to obtain the COP position time series. The subject stands on the force plate during the experiment. The collected COP position time series is sent to personal computer through USB.

The sampling frequencies of both sensors are set to 1000 Hz. And the sample synchronization of two-sensor system is via a trigger of rising edge.

### 2.3. Experimental Method

After measuring and recording the basic information (height and weight), the angle sensor was positioned at the ankle, knee, and hip joints. Subjects stood barefoot on a static platform, with hands naturally at their sides. Their two feet were apart at their shoulder-width, and so on (as shown in Figure 1). They were asked not to lift foot or swing arms, if possible using only the ankle, knee, and hip joints to adjust their balancing postures. Subjects were asked to stand upright in eyes-closed (EC) and eyes-open (EO) condition, respectively, during 100 s.

## 3. Metric Algorithm

### 3.1. Filtering

The time series obtained in this experiment was finite and noisy. A band pass digital filter with a range frequency of 0.1–5.0 Hz was applied in this study. On the one hand, no discernable spectral peaks were found above 5 Hz for normal subjects [3, 10]. On the other hand, COP always migrates in special pattern, which is fast or slow continuous displacement around the average position of COP [10, 11]. The raw time series captured from the angle sensors and the force plate were pretreated in the software environment of MATLAB 2010b. Data of the two typical subjects are shown in Section 4.

### 3.2. Reconstruction of Phase Space of Multivariate System

For computing the LLE, the first step is to carry out a reasonable reconstruction of the phase space of the system [12]. The reconstructed phase space from the dynamical data must preserve the invariant characteristics of the original unknown multivariate system. Because the data in experiment is finite and noisy, choice of delay time is important in the reconstruction of the attractor. As in the case of one-dimensional discrete time series, the coordinate delay method is used by embedding time series variable delay to reconstruct the phase space of a nonlinear system. Supposing that the human body is a multivariate system, we then have an *M*-dimensional time series from the electrical goniometer system: {*x*}_*n*=1_^*N*^ = {*x*_1,*n*_, *x*_2,*n*_,…,*x*_*M*,*n*_}_*n*=1_^*M*^. The phase space reconstruction can be described by

(1) Vn=x1,n,x1,n−τ1,…,x1,n−m1−1τ1;x2,n,x2,n−τ2,…,x2,n−m2−1τ2;…;xM,n,xM,n−τM,…,xM,n−mM−1τMn=J0,J0+1,…,N;  J0=max⁡1≤i≤M⁡mi−1τi+1,

where *τ*_*i*_ and *m*_*i*_ (*i* = 1,2,…, *M*) are the time delays and the embedding dimensions, respectively. Following Takens' delay-embedding theorem, there exists in the generic case a function *f* : *ℜ* ^*d*^ → *ℜ* ^*d*^ (*d* = ∑_*I*=1_^*M*^ *d*_*i*_),

(2) $$\begin{array}{c} V_{n+1}=f\left(\;V_{n}\;\right). \end{array}$$

Time delay *τ*_*i*_ affects the quality of the reconstructed phase space. If the selection of each time delay *τ*_*i*_ is reasonable and each embedding dimension *m*_*i*_ is sufficiently large, the geometrical characteristics of the strange attractor in the reconstructed space are equivalent to the original state space [13].

Using mutual information [14, 15], the time delay of each variable of the time series can be solved. Based on information theory, the mutual information between time series *X* and its delay time series *X*_*τ*_ can be derived:

(3) $$\begin{array}{c} I\left(\;\tau \;\right)=I\left(\;X,X_{\tau }\;\right)=H\left(\;X\;\right)+H\left(\;X_{\tau }\;\right)-H\left(\;X,X_{\tau }\;\right). \end{array}$$

We compute the mutual information *I*(*τ*_*i*_) by the histogram-based statistic estimator [15], and *τ*_*i*_, which makes *I*(*τ*_*i*_) to the first local minimum point, is regarded as the delay time of *i*th variable. By calculating time series data of all subjects, reasonable reconstructing time lag can be obtained (the average time lag of 36 subjects is *τ*_ankle_ = 17 ± 7.3, *τ*_knee_ = 21 ± 5.8, and *τ*_hip_ = 19 ± 8.3).

In 1998, Cao et al. proposed a method for obtaining the embedding dimension *m*, which was named as the prediction error method [8]. By using the continuity of *f* in (2), this method assumes to choose the embedding dimension *m* reasonably; if the points *V*_*n*_ and *V*_*j*_ in the reconstructed attractor space are sufficiently close, then *x*(*i*, *n* + 1) and *x*(*i*, *j* + 1) will be relatively close. We consider the problem of finding the embedding dimensions of *f*. We put the determined time delays vector *τ* and randomly chosen embedding dimension vector *m* into Formula (2). For any given set of dimensions, the reconstruction phase space results ${\tilde{V}}_{n}$ of the initial parameters are obtained, where *n* = max⁡_*l*<*i*<*M*_⁡(*d*_*i*_ − 1) + 1,…, *N*. Suppose it has a nearest neighbour ${\tilde{V}}_{j}$. Then the initial distance by Euclidean norm is

(4) $$\begin{array}{c} d_{j}\left(\;0\;\right)=\min\left\|\;V_{n}-V_{j}\;\right\|. \end{array}$$

The prediction error function is defined as

(5) Em1,m2,…,mM=1N−J0+1∑n=J0Nx1,n+1−x1,μn+1,J0=max⁡l≤i≤M⁡mi−1τi+1.

The error measure *E* depends on choosing embedding dimensions *m*_1_, *m*_2_,…, *m*_*M*_ which minimize *E*. The initial embedding dimension vector *m*_1_, *m*_2_,…, *m*_*M*_ is chosen randomly, so the *E*(*m*_1_, *m*_2_,…, *m*_*M*_) gained will be of a greater value. When the iteration proceeds, the vector *m*_1_, *m*_2_,…, *m*_*M*_ will be constantly revised and will approach a more reasonable range, with decreasing *E* values. By the method of Cao, we chose the vector *m*_1_, *m*_2_,…, *m*_*M*_, which makes *E* to the first local minimum,

(6) m1,m2,…,mM=arg⁡min⁡⁡Em1,m2,…,mM ∣ m1,m2,…,mM∈ZM,∑i=1Mmi≠0,

where *Z* ^*M*^ = ∏_*I*=1_^*M*^ *Z* represents all nonnegative integers. By calculating the time series data of all subjects, reasonable embedding dimensions *m*_ankle_, *m*_knee_, and *m*_hip_ can be obtained (the average embedding dimensions of 36 subjects are *m*_ankle_ = 4.02 ± 0.94, *m*_knee_ = 4.17 ± 0.73, and *m*_hip_ = 5.03 ± 0.81). Since the optimal embedding dimension vector is also unique to one dynamical system, the corresponding optimal reconstructing parameters sets [*m*_ankle_, *m*_knee_, *m*_hip_] and [*τ*_ankle_, *τ*_knee_, *τ*_hip_] should be applied to calculate the MLLE for the time series of one multivariate dynamical system.

### 3.3. Determinism Test and Stationarity Test

In reality, because the time series is noisy, the typically nonlinear parameter cannot always correctly quantify the dynamics of the original dynamic system. Therefore, two confirmatory tests (determinism test and stationarity test) need to be carried out to prove whether systems of human standing belong to dynamic system. If the two tests are positive, we could then proceed to calculate the MLLE of the multivariate dynamic system [16–18].

The determinism test, which is based on a correct reconstruction of the attractor, enables us to measure average directional vectors in the coarse-grained embedding space [19]. The embedding space should be coarse-grained into equally sized boxes. Each pass *l* of the trajectory through the *k*th box can be regarded as a unit vector *e*_*l*_, and their directions are approximated by the points where the particular pass enters and leaves the box. Their average directional vector *V*_*k*_ of the unit vector through the *k*th box is defined by

(7) $$\begin{array}{c} V_{k}=\frac{1}{C}\overset{C}{\underset{p=1}{\sum }}e_{l}, \end{array}$$

where *C* is the total of all passes in box *k*. Therefore, an approximate direction for the vector field can be obtained by all occupied boxes. If the data set sources from a deterministic dynamic process as well as a fine enough coarse-grained partitioning, the vector *e*_*l*_ inside one box would nearly not cross. Since each crossing will decrease the size of the average vector *V*_*k*_, the average length of all directional vectors will be 1 for a deterministic process, while for a random process it will be 0.

In order to verify whether the studied sway is from a stationarity process, stationarity test is evaluated [16, 20]. To perform this method, the time series (20000 points) is divided into *h* (*h* = 100) short nonoverlapping segments; therefore, a cross-prediction error (*δ*_*ij*_) statistic is calculated for *h* ^2^ possible combinations. We obtain a very sensitive statistic capable of detecting minute changes in dynamics and thus a very powerful probe for stationarity. If *δ*_*ij*_ of each combination is not much larger than the average, the examined time series can be considered to be from a stationary system.

**Figure 2:**
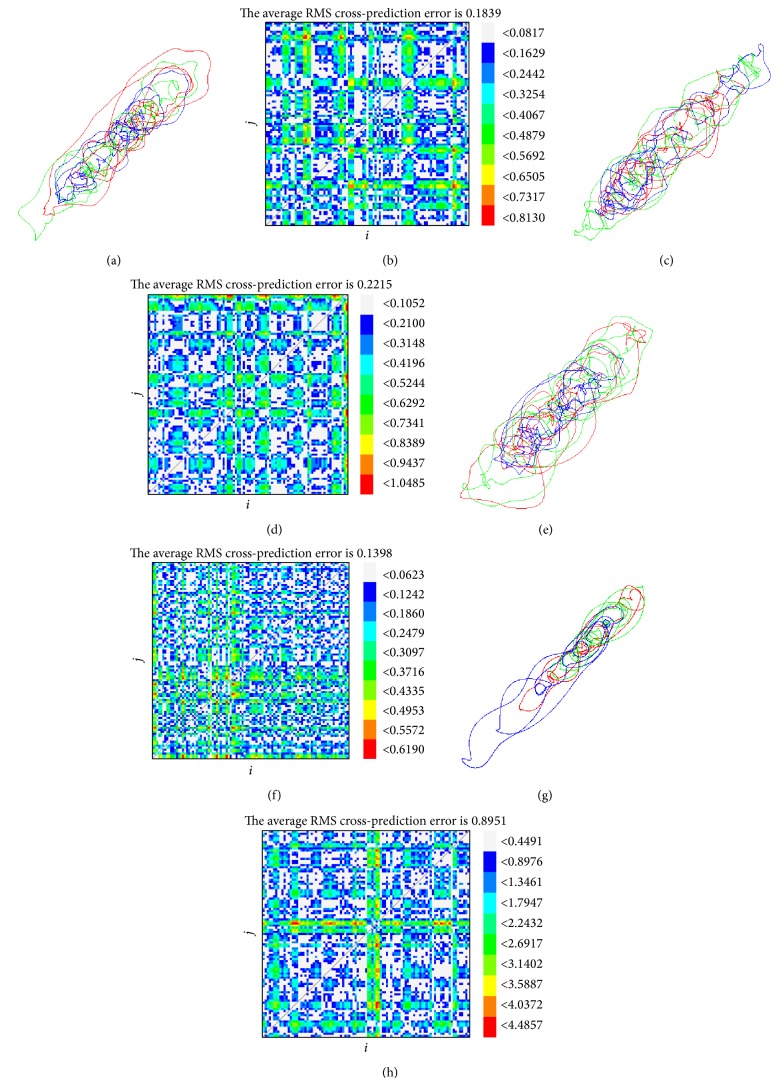
The results of the determinism test and stationarity test for the acceleration time series of a typical subject. (a) and (b) are the results of the hip, (c) and (d) are the results of the knee, (e) and (f) are the results of the ankle, and (g) and (h) are the results of COP in AP. (a), (c), (e), and (g) are the embedding space, and (b), (d), (f), and (h) are the average cross-prediction error for all the possible combinations of *i* and *j*. The average values of all *δ*_*ij*_ are 0.1839, 0.2215, 0.1398, and 0.8951 (for the time series of hip, knee, ankle, and COP in AP, resp.).

In this section, the data set of joints and COP in AP from a typical subject is evaluated by the determinism test and stationarity test. The determinism factors are 0.8385 (time series of ankle angle, *τ* = 20 and *m* = 3), 0.8202 (time series of knee angle, *τ* = 22 and *m* = 4), 0.8330 (time series of hip angle, *τ* = 24 and *m* = 3), and 0.8612 (time series of hip angle, *τ* = 30 and *m* = 4). The pertained to embedding spaces are shown in Figures 2(a), 2(c), 2(e), and 2(g). This clearly confirms the deterministic nature of human balance system. The average cross-prediction error for all possible combinations of *i* and *j* is given in Figures 2(b), 2(d), 2(f), and 2(h). The average values of all *δ*_*ij*_ are 0.1839, 0.2215, 0.1398, and 0.8951 (for the time series of hip, knee, ankle, and COP in AP, resp.). Since each maximal cross-prediction error is not significantly larger than the average, the studied time series are clearly stationary.

### 3.4. Algorithm of Multivariate LLE

Reference [9] proposed the multivariate largest Lyapunov exponent (MLLE) calculation method. In the multivariate phase space, supposing that each point *V*_*j*_ has a nearest neighbor point $V_{\hat{j}}$, thus, there must be a short separation between these two points in order to ensure that the two points are running along different tracks. The separation interval is defined as *w* = max⁡⁡(*T*_ankle_, *T*_knee_, *T*_hip_), and *T*_ankle_, *T*_knee_, *T*_hip_ denotes the mean period of each time series, and a unique *w* is set at 1000 in this work. The distance between *V*_*j*_ and $V_{\hat{j}}$ is defined as *d*_*j*_(0):

(8) $$\begin{array}{c} d_{j}\left(\;0\;\right)=\min\left\|\;V_{j}-V_{\hat{j}}\;\right\|,\;\left|j-\hat{j}\right|>w. \end{array}$$

For each point *V*_*j*_ in the phase space, we can calculate the distance to its nearest neighbour point after the evolution of the *k* steps:

(9) $$\begin{array}{c} d_{j}\left(\;k\;\right)=\min\left\|\;V_{\left(j+1\right)}-V_{\left(\hat{j}+k\right)}\;\right\|, \end{array}$$

where *k* = *J*_0_, *J*_0_ + 1,…, *N*. Suppose that the rate of divergence of *V*_*j*_ and its nearest neighbour point $V_{\hat{j}}$ is the LLE, which is represented by *γ*; thus *d*_*j*_(*k*) = *d*_*j*_(0) × *e* ^*γ*(*k*·Δ*t*)^. Taking the logarithm of both sides, we obtain ln⁡*d*_*j*_(*k*) = ln⁡*d*_*j*_(0) × *γ*(*k* · Δ*t*). By least squares, the curve 〈ln⁡*d*_*j*_(*k*)〉 versus *k* · Δ*t* can be fitted, which is the LLE value:

(10) $$\begin{array}{c} \gamma =\frac{1}{\sum k^{2}}\left[\;k\times \frac{1}{h\Delta t}\overset{h}{\underset{j=1}{\sum }}\ln d_{j}\left(\;k\;\right)\;\right], \end{array}$$

where *h* is the number of *d*_*j*_(*k*).

## 4. Results

### 4.1. Power Spectral Density Analysis

**Figure 3:**
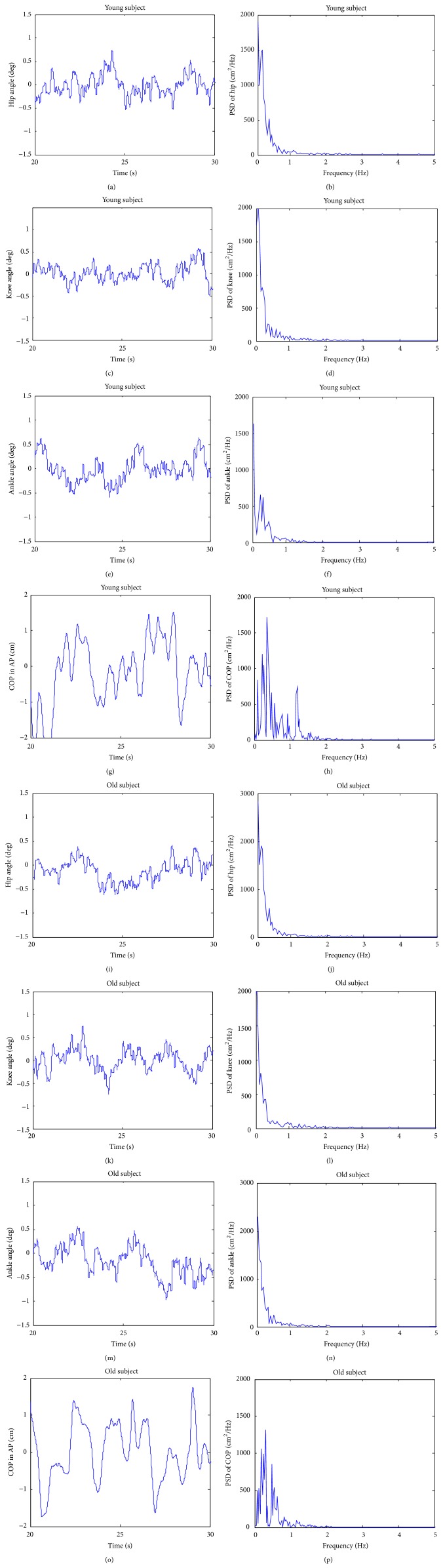
The results of PSD analysis. A PSD analysis of the frequency domain was carried out. The results of one typical young subject and one typical old subject are plotted, where ten seconds of the four typical traces obtained for COP and the sagittal kinematics is on the left, together with the corresponding PSD on the right. (a), (c), (e), (g), (i), (k), (m), and (o) are the time series and (b), (d), (f), (h), (j), (l), (n), and (p) are their corresponding PSD plots.

In order to compare the method with the traditional method, a power spectral density (PSD) analysis was carried out on each time series of the subjects. The results of two typical subjects are plotted in Figure 3, where ten seconds of the four typical traces obtained for COP and the sagittal kinematics is on the left, together with the corresponding PSD on the right.

A one-way analysis of variance (ANOVA) was conducted by SPSS 19.0 (SPSS, Inc., Chicago, IL) to determine if there was statistical significance between different groups under the EO or EC condition. The level of significant difference was set at *p* < 0.05.

The average PSD of COP signal in different frequency bands, which were defined as 0.1–0.5 Hz, 0.5–1 Hz, 1–1.5 Hz, and 1.5–2 Hz, were analysed. The old group showed a smaller average PSD than that of the young group under the EC condition in all frequency bands. However, there is no significant difference in average PSD (*p* > 0.05, Table 1). The eyes closing increases the average PSD values of frequency bands 0.1–0.5 Hz and 1–1.5 Hz in the old group (*p* < 0.05, Table 1). In the young group, PSD values under the EC condition are higher than that under the EO condition (Table 1). However, the variances of PSD under the EC condition in both groups are much larger, except the average PSD values of frequency bands 0.1–0.5 Hz (*p* < 0.05, Table 1). In addition, there is no significant difference in average PSD between both groups in the two conditions (*p* > 0.05, Table 1).

### 4.2. Results of MLLE Values

**Figure 4:**
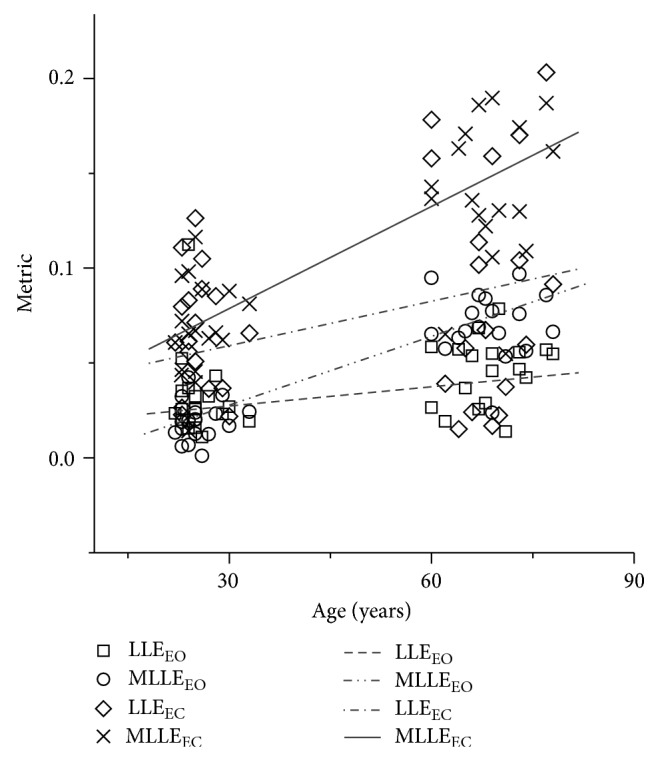
The results of LLE and MLLE. Abscissa denotes the actual age of the subjects; vertical axis denotes the dimensionless exponent; all metric values of each method are fitted by linear curves. The exponents LLEEC and LLEEO denote the values from the COP data in CE and OE conditions, respectively; MLLEEC and MLLEEO denote the values from the joint angle data in CE and OE conditions, respectively.

With the experimental data, multijointed body MLLE values were obtained by Function (10). Those values included the results under EO condition and EC condition. In order to carry out a comparison with previous conclusions obtained from one-dimensional COP data, the method presented in [6, 21] was performed. All of the human standing balance ability measurement results are shown in Figure 4. A one-way analysis of variance (ANOVA) was conducted to determine if there was statistical significance between different groups under EO or EC condition in MLLE values or LLE values.

In Figure 4, the abscissa is the actual age of the subjects, and the vertical axis is the dimensionless exponent; the exponents LLE_EC_ and LLE_EO_ denote the numerical metrics based on the COP data in EC and EO conditions, respectively; MLLE_EC_ and MLLE_EO_ denote the numerical metric based on the joint angle data in EC and EO conditions, respectively. The numerical metric of four methods is fitted with a linear curve.

The subjects of different ages are mainly concentrated in two regions of the abscissa. According to the visual and age conditions, there is a significant difference in the distributions of the two metrics' values. Intuitively, a greater slope of the fitted curve indicates that the metric has a bigger advantage in distinguishing different individuals in the age group. The fitted curves in Figure 4 show the effect of each metric: MLLE_EC_ > MLLE_EO_ > LLE_EC_ > LLE_EO_. There is significant difference between two groups in EC conditions by the metric MLLE_EC_ (*p* < 0.05).

**Figure 5:**
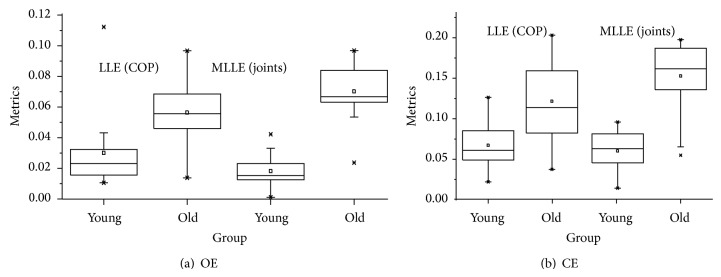
The results of the boxplot. (a) Results in EO condition. (b) Results in EC condition. The results were statistically analysed by a boxplot: (a) the statistical results in the EO condition; (b) the statistical results in EC condition.

In Figure 5, the results were statistically analysed by a boxplot: (a) the statistical results in EO condition; (b) the statistical results in EC condition. In EO condition, although the values of LLE and MLLE can distinguish individuals into two groups of different ages, there are more evident differences in the MLLE mean between the groups, which indicate that the MLLE algorithm has better distinguishing effects. Meanwhile, the LLE values for the young group have greater volatility, which indicates that the algorithm based on COP data does not give a reliable evaluation for some individuals.

In the boxplots of Figure 5(b), mean values for most subjects in the EC condition increase compared with those values in the EO condition, which is good for distinguishing the different subjects. Also, we can see that the upper and lower quartiles are enlarged, which indicates that the mean difference between the groups is still large but does not affect the distinction between the two groups.

## 5. Discussion

The LLE based on the COP time series has been discussed as a data analysis indicator by many researchers [1, 3, 21]. However, in practical problems, it cannot guarantee that any given one-dimensional time series is sufficient to reconstruct the dynamical characteristics of chaotic systems [8, 9]. There is a complicated coupling relationship in the body structure. Stable standing balance depends on the ability of CNS to control all body segments. This capability seems not to be fully expressed by the motion data of a single segment, so the one joint angle or COP data can only contain partially dynamical characteristics of chaotic systems. In order to compensate for the lack of a one-dimensional time series LLE, the approach based on multidimensional time series is proposed. Theoretically, the metric of MLLE represents the special average of the *M*-dimensional movement of attractor trajectories of different body segments.

In Table 1, the ANOVA results indicate that vision plays an important role in maintaining the balance. As well as in Figures 4 and 5, it can be seen that the divergence metrics (LLE and MLLE) of the attractor show a significant increase. Both trends indicate that the human proprioceptive system is reduced with the increment of age, which is the same as the result ofJiang and Hidenori [22, 23], and maintaining body balance is more dependent on visual information feedback.

An MLLE value has a higher accuracy to distinguish subjects from different age groups under the EC condition. Although there is an extreme outlying MLLE value in the boxplot, the overall mean and variance of the results clearly illustrate the effectiveness of this approach. CNS uses various control strategies to reduce the complexity of posture control [24]. Different selections of control strategies diversify the individuals' dynamic process. This may be the reason for the failure to describe the characteristic of the subject.

The results in Table 1 illustrate that the traditional PSD method is not satisfactory to express intergroup differences. This may be caused by many reasons. The pretreatment method for the original data, such as different filter parameters of the pass band, will impact the value of PSD, and the results of frequency domain analysis are apparently impacted by the high-pass cutoff frequency. If the experimental data are recorded for a long time, several types of human movement COP would be found [10, 11]. However, in this research, the experimental data were recorded for only 100 seconds. A low-frequency COP sway trace was generally considered as low-frequency errors from the sensor. In order to ensure the stability of the data, a low cutoff frequency of 0.1 Hz was applied.

The Lyapunov exponent spectrum of the COP signal has been applied to characterize the human standing activity as chaotic and as an aid to identify arrhythmias. The minimal distance to define these two points of the phase space is *w*, the range of which has been given in Section 3.4. In order to obtain a uniform evaluation and to avoid the interference of the evaluation of different parameters, *w* was set at 1000, which was within a reasonable range. Different working parameters of *w* may lead to different conclusions about the same data [25]. If we change the *w* value, the metric would be inaccurate or even fail to assess human standing ability. A sensitivity analysis of parameter *w* will be carried out by using the Lyapunov exponent spectrum in our future work.

## 6. Conclusion

Chaos theory has been used to explain complicated temporal behavior in many research areas. In this study, human standing balance ability is quantified by MLLE. Compared with the existing method, the metric based on multivariate largest Lyapunov exponent has a higher degree of differentiation in differentiating balance in eyes-closed conditions. The MLLE value reflects the overall coordination between multisegment movements. Individuals of different ages can be distinguished by their MLLE values to some extent. The stability of human standing has reduced trend with the increment of age.

## Figures and Tables

**Table 1:** Average PSD values for COP in AP of all subjects under different eyes conditions. The bands of frequency area are defined as 0.1–0.5 Hz, 0.5–1 Hz, 1–1.5 Hz, and 1.5–2 Hz.

Eyes condition	0.1–0.5 Hz		0.5–1.0 Hz		1.0–1.5 Hz		1.5–2.0 Hz	
	Young	Old	Young	Old	Young	Old	Young	Old
EO	760 ± 220^∗^	742 ± 280	130 ± 50	124 ± 47^∗^	43 ± 18	37 ± 19^∗^	36 ± 15	33 ± 14
EC	828 ± 350^∗^	793 ± 310	104 ± 32	88 ± 39^∗^	37 ± 22	28 ± 8^∗^	31 ± 17	29 ± 11

^∗^The significant difference between different eyes conditions (*p* < 0.05).
